# Supplementary material for: Adaptive laboratory evolution of microbial co‐cultures for improved metabolite secretion
Source: Mol Syst Biol. 2021 Aug 9;17(8):e10189. doi: 10.15252/msb.202010189 (PMC8351387; doi:10.15252/msb.202010189)
Supplement: Supplementary file 1 — Appendix [file MSB-17-e10189-s001.pdf]

Appendix for:

Adaptive laboratory evolution of microbial co-cultures for improved metabolite secretion

Dimitrios Konstantinidis[1,2], Filipa Pereira[1], Eva-Maria Geissen[1], Kristina Grkovska[1], Eleni Kafkia[1,3], Paula Jouhten[4], Yongkyu Kim[1,5], Saravanan Devendran[1], Michael Zimmermann[1], Kiran Raosaheb Patil[1,3*]

[1] Structural and Computational Biology Unit, European Molecular Biology Laboratory, Heidelberg, Germany
[2] Faculty of Biosciences, Heidelberg University, Heidelberg, Germany
[3] Medical Research Council Toxicology Unit, Cambridge, United Kingdom
[4] VTT Technical Research Centre of Finland Ltd, Espoo, Finland
[5] Present address: Brain Research Institute, Korea Institute of Research and Technology, Seoul, South Korea
*correspondence to: kp533@cam.ac.uk

Contents:

Appendix Figure S1. Effect of medium conditioned by *L. plantarum* on the fitness of evolved and parental riboflavin auxotrophic yeast strains.

Appendix Figure S2. Co-evolution for the increased production of folate.

Appendix Figure S3. ALE experiment performed either with bacterial monocultures or with co-cultures of different bacterial species and *S. cerevisiae*.

Appendix Table S1. Summary of the flavins producing phenotype.

Appendix Table S2. Shortlist of identified mutations in the evolved *L. plantarum* strains after the co-evolution ALE for increased riboflavin production.

Appendix Table S3. List of the supplemented amino acids.

Appendix Table S4. List of plasmids and primers used in the present study.

Appendix Table S5. Description of the UPLC method used for detection of vitamins.

Appendix Table S6. Model inputs.

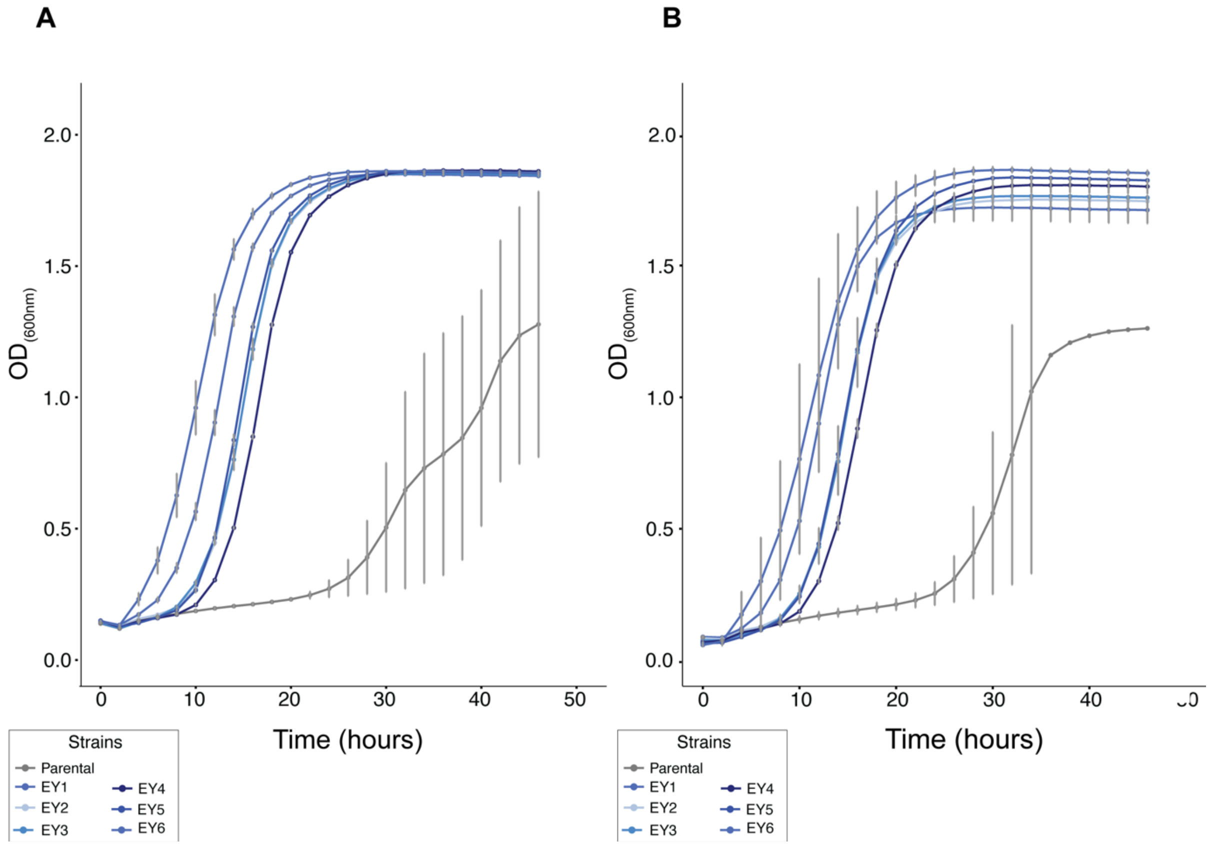

Appendix Figure S1. Effect of medium conditioned by *L. plantarum* on the fitness of evolved and parental riboflavin auxotrophic yeast strains. A) Growth kinetics of the parental Δ*rib4:rib5 S. cerevisiae* and 6 evolved yeast isolates in conditioned medium of the parental *L. plantarum* strain (n= 3 biologically independent samples; data are presented as means ± s.d – grey bars). **B)** Growth kinetics of the parental *Δrib4:rib5 S. cerevisiae* and 6 evolved yeast isolates in conditioned medium of the evolved *L. plantarum* strain E6 (n= 3 biologically independent samples; data are presented as means ± s.d – grey bars). In the figure the average value of biological triplicates is represented and the standard deviation is represented as a grey bar.

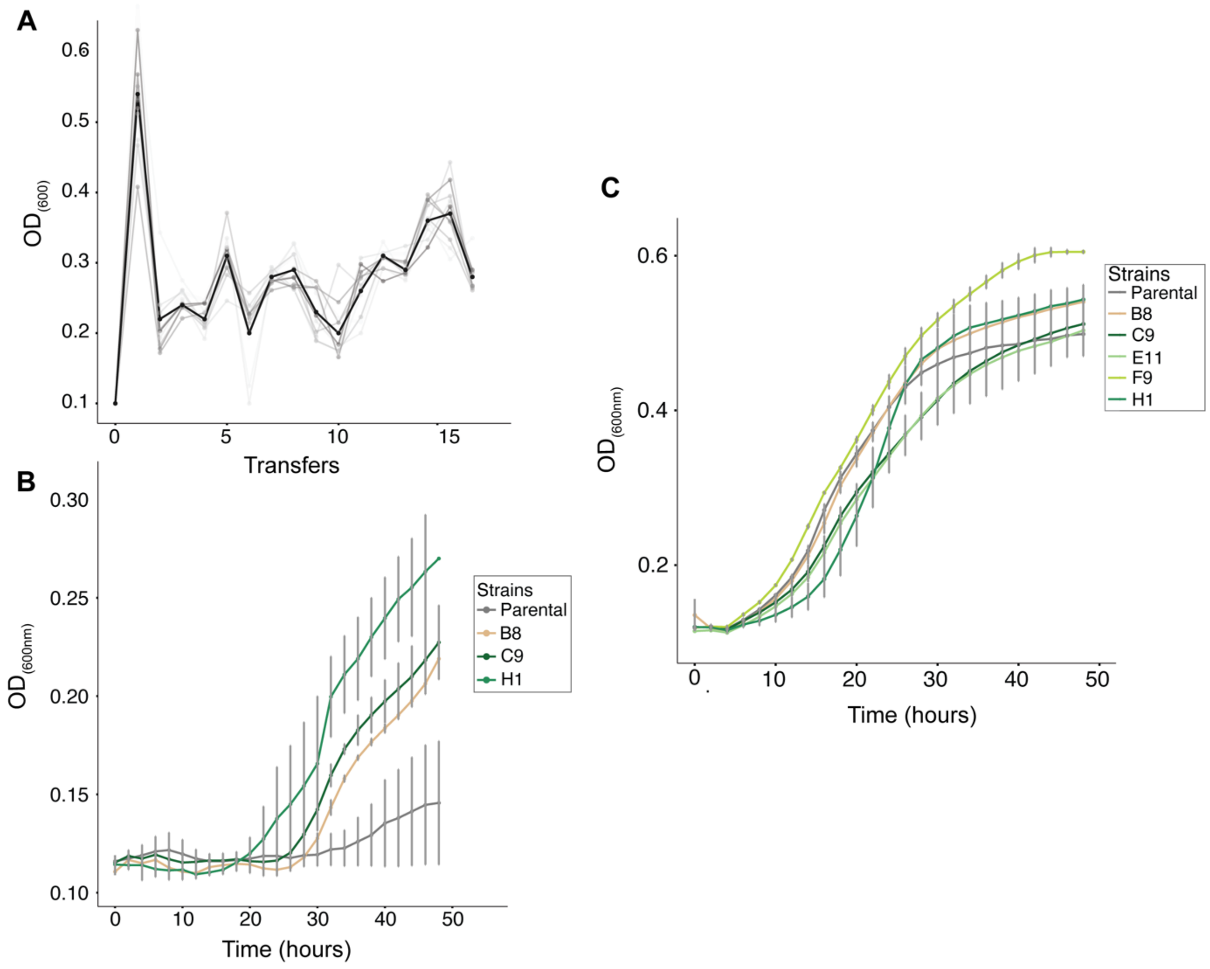

Appendix Figure S2. Co-evolution for the increased production of folate. A) Twelve populations (*L. plantarum* + Δ*abz1 S. cerevisiae* strain), deriving from the same parental cultures, were evolved in parallel in 96well plates. The growth of each community improved similarly for every replicate, based on Optical Density measurements. In the figure are the dark black line represents the mean OD_{600} values of the 12 populations. **B)** Growth kinetics of the parental and evolved bacteria strains in conditioned medium from wild-type *S. cerevisiae* (n= 3 biologically independent samples; data are presented as means ± s.d – grey bars). **C)** Growth kinetics of the parental Δ*abz1 S. cerevisiae* strain in conditioned medium of the parental and evolved bacteria strains (n= 3 biologically independent samples; data are presented as means ± s.d – grey bars).

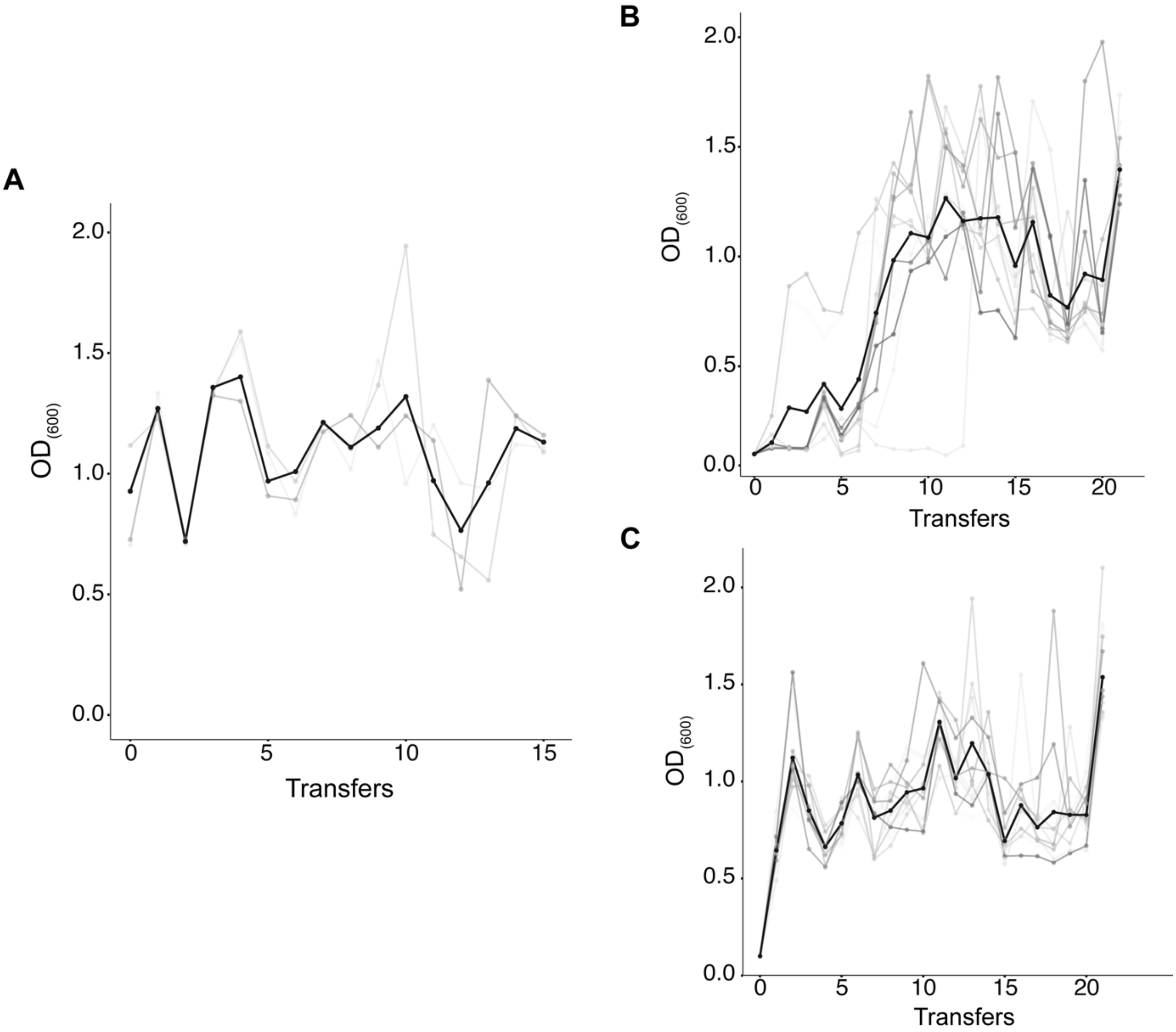

Appendix Figure S3. ALE experiment performed either with bacterial monocultures or with co-cultures of different bacterial species and *S. cerevisiae*. A) Four *L. plantarum* monocultures deriving from the same parental culture were evolved in parallel in 96well plates. The growth of each community improved similarly for every replicate, based on Optical Density measurements. In the figure are the dark black line represents the mean OD_{600} values of the monocultures. **B)** Twelve populations (*L. lactis* + *Δrib4:rib5 S. cerevisiae* strain) deriving from the same parental cultures were evolved in parallel in 96well plates. The growth of each community improved similarly for every replicate, based on Optical Density measurements. In the figure are the dark black line represents the mean OD_{600} values of the 12 populations. **C)** Twelve populations (*B. casei* + *Δrib4:rib5 S. cerevisiae* strain) deriving from the same parental cultures were evolved in parallel in 96well plates. The growth of each community improved similarly for every replicate, based on Optical Density measurements. In the figure are the dark black line represents the mean OD_{600} values of the 12 populations.

Appendix Table S1. Summary of the flavins producing phenotype. The values of the parental strain and six evolved isolates were determined with 3 different approaches.

			Parental	B4	C2	D5	E6	G7
Fluorescence assay			5213 ±132.69	22317	16174	2920	28684	23394
UPLC*	Extracellular	Riboflavin (AUC)	84.96 ±5.38	96.96 ±5.64	99.15 ±7.96	86.3 ±13.8	109.39 ±16.44	99.91 ±6.90
		FAD (AUC)	0.29 ±0.01	0.30 ±0.07	0.27 ±0.17	0.28 ±0.1	0.68 ±0.01	0.43 ±0.02
	Intracellular	Riboflavin (AUC)	0.22 ±0.01	0.73 ±0.09	0.62 ±0.16	0.25 ±0.03	0.40 ±0.09	0.39 ±0.10
		FMN (AUC)	0.02 ±0.01	0.08 ±0.02	0.07 ±0.01	0.03 ±0.01	0.06 ±0.02	0.04 ±0.01
LCMS	Extracellular	Riboflavin (ng/mL)	42 ±16.4	134 ±61.1	60.9 ±20.7	N/A	426 ±208.7	18.2 ±3.2
		FAD (ng/mL)	0.015 ±0.003	0.015 ±0.001	0.024 ±0.002	N/A	0.018 ±0.002	0.029 ±0.006
	Intracellular	Riboflavin (ng/mL)	10.3 ±1.7	144 ±55.1	11.1 ±6.7	N/A	27 ±14.9	15 ±16.6
		FMN (ng/mL)	5.2 ±0.9	30.5 ±14	8.9 ±7.9	N/A	20.8 ±12.7	13 ±10.6
		FAD (ng/mL)	2.2 ±0.7	5.8 ±0.9	7.8 ±6.4	N/A	13.7 ±6	9.2 ±7.7

Fluorescence assay: parental value was calculated from 5 independent biological replicates (± standard deviation), each isolated strain was measured once.
UPLC data was calculated using 3 independent biological replicate (± standard deviation).
* Area Under the Curve (AUC) of targeted metabolite (Riboflavin, FMN or FAD) normalized by the AUC of the internal standard.
LCMS data was calculated using 3 independent biological replicate (± standard deviation).
N/A : Measurement not available.

Appendix Table S2. Shortlist of identified mutations in the evolved *L. plantarum* strains after the co-evolution ALE for increased riboflavin production.

Isolate	Gene	Description	Mutation	Position	Frequency (%)	Annotation
C2	BGV74_00535 →	MarR family transcriptional regulator	2 bp→TG	116,190	100	coding (183184/474 nt)
E6	BGV74_00535 →	MarR family transcriptional regulator	T→C	116,343	100	G112G (GGT→GGC)
G7	BGV74_00535 →	MarR family transcriptional regulator	A→C	116,193	100	P62P (CCA→CCC)
B4	BGV74_02540 →	transcription antiterminator BglG	G→T	533,100	100	D172Y (GAT→TAT)
C2	BGV74_03220 →	cell surface protein	A→T	683,919	100	D1561V (GAT→GTT)
G7	BGV74_03220 →	cell surface protein	A→G	683,919	100	D1561G (GAT→GGT)
C2	BGV74_03655 →	transcription antiterminator LicT	A→T	778,261	100	Y201F (TAT→TTT)
G7	BGV74_04555 → / → BGV74_04560	amino acid permease/ECF transporter S component	A→G	968,444	100	intergenic (+56/65)
E6	BGV74_04865 →	dTDPglucose 4,6dehydratase	A→G	1,039,415	100	E237E (GAA→GAG)
C2	BGV74_04925 →	transporter	A→C	1,051,199	100	I487L (ATT→CTT)
C2	BGV74_05060 →	exopolysaccharide biosynthesis protein	A→C	1,076,641	100	H123P (CAT→CCT)
C2	BGV74_05065 ←	AraC family transcriptional regulator	G→C	1,077,186	100	Q246E (CAG→GAG)
G7	BGV74_05075 →	MarR family transcriptional regulator	G→A	1,082,031	100	A128T (GCT→ACC)
G7	BGV74_05085 ←	multidrug MFS transporter	G→A	1,084,130	100	Y206Y (TAC→TAT)
C2	BGV74_05220 →	peptide ABC transporter permease	A→G	1,112,955	100	S44G (AGC→GGC)
B4	BGV74_05465 →	cell shape determining protein MreB	C→T	1,161,638	100	pseudogene (4245/8007 nt)
E6	BGV74_05465 →	cell shapedetermining protein MreB	C→G	1,161,173	100	pseudogene (3780/8007 nt)
E6	BGV74_05465 →	cell shapedetermining protein MreB	C→T	1,161,632	100	pseudogene (4239/8007 nt)

C2	BGV74_06015 →	ABC transporter permease	C→A	1,290,355	100	R249S (CGT→AGT)
G7	BGV74_07265 ← / ← BGV74_07270	iron ABC transporter ATPbinding protein/carboxypeptidase	3 bp→TCG	1,544,959	100	intergenic (160/+23)
B4	BGV74_07495 → / ← BGV74_07500	cyclic nucleotidebinding protein/DUF368 domaincontaining protein	TTCAACG GCA	1,588,790	100	intergenic (+96/+30)
E6	BGV74_07605 ←	LysR family transcriptional regulator	G→A	1,613,143	100	P166S (CCA→TCA)
G7	BGV74_08665 ← / ← BGV74_08670	hypothetical protein/transcriptional regulator	C→A	1,850,027	100	intergenic (262/+151)
C2	BGV74_10200 ←	multidrug ABC transporter ATPbinding protein	2 bp→AG	2,145,443	100	coding (545546/189 0 nt)
B4	BGV74_10430 ←	transcriptional regulator	G→T	2,185,316	100	N109K (AAC→AAA)
B4	BGV74_10430 ←	transcriptional regulator	2 bp→CT	2,185,318	100	coding (324325/513 nt)
B4	BGV74_10450 → / → BGV74_10455	ImmA/IrrE family metalloendopeptidase/hypothetic al protein	A→C	2,187,404	100	intergenic (+4/19)
G7	BGV74_10565 ←	mucusbinding protein	T→C	2,200,103	100	T795T (ACA→ACG)
B4	BGV74_12305 ←	cell surface protein	A→T	2,596,574	100	S347S (TCT→TCA)
C2	BGV74_12675 ←	glutamate/gammaaminobutyrate family transporter YjeM	2 bp→AC	2,672,890	100	coding (961962/148 5 nt)
B4	BGV74_12685 → / ← BGV74_12690	1,3propanediol dehydrogenase/arylalcohol dehydrogenase	C→T	2,677,088	100	intergenic (+1077/+153)
G7	BGV74_12685 → / ← BGV74_12690	1,3propanediol dehydrogenase/arylalcohol dehydrogenase	C→T	2,677,088	100	intergenic (+1077/+153)
C2	BGV74_12995 →	fumarate reductase	T→G	2,754,473	100	F292C (TTC→TGC)
C2	BGV74_14110 ←	TetR family transcriptional regulator	2 bp→CT	2,990,663	100	coding (221222/639 nt)

Appendix Table S3. List of the supplemented amino acids. The name and the concentration of amino acids, that need to be supplemented in CDM35 to support the growth of LAB in monoculture.

Amino acid	Concentration (g/L)
L-Glutamic acid	15
L-Phenylalanine	10
L-Proline	17.5
L-Asparagine	12.5
L-Aspartic acid	10.5
L-Glutamine	15
L-Serine	12.5
L-Threonine	12.5
L-Cysteine	5
L-Alanine	10
Glycine	7.5
L-Lysine	10
L-Tryptophan	5

Appendix Table S4. List of plasmids and primers used in the present study.

Plasmid	Gene	Primers (5'-3')
puG6	*RIB4*	Forward:<br>GCAGTATAACGCAGTATAACGCAGTATAACGCAGTAGCGACATGGAGGCCCAGAA<br>Reverse:<br>GCGCTTATTCAAAAAGCATTTTTACCGAACTTAACTCGACACTGGATGGCGGCGT
puG32	*RIB5*	Forward:<br>ATGTTTACTGGTATTGTAGAATGCATGGGGACTGTAGCGACATGGAGGCCCAGAA<br>Reverse:<br>GGTAGTTTCTAACCTTCTCCTCGATAATGTTTGAGTCGACACTGGATGGCGGCGT
puG6	*ABZ1*	Forward:<br>ATCACCTACACTATCTTCAGCAAGG<br>Reverse:<br>CTTTAGGTACGGTTGTTGTCATCTT

Appendix Table S5. Description of the UPLC method used for detection of vitamins.

**Time (minutes)**	**Flow (mL/min)**	**Acetonitrile 100% + 10mM Ammonium acetate, pH 9**	**Acetonitrile 50% + 10mM Ammonium acetate, pH 9**
0-3	0.35	10	90
3-5	0.35	70	30
5-7	0.35	10	90
7-8	0.35	19	90

Appendix Table S6. Model inputs. Their description and values used for simulations in Figure EV3 (default value)

input variable	description	default value
initial_number_yeast	The number of yeast cells for the initial simulation setup.	50
initial_ratio	The initial ratio between bacteria and yeast cells. Determines together with the input initial_number_yeast the total initial amount of bacteria.	10
initial_number_mutant	Initial number of mutant bacterial agents. This number is subtracted from the total number of bacteria to yield the number of wild type bacterial agents	1

Inputs determining the physiology of agents		
All bacterial agents		
init_energy_bacteria	The number of energy units per bacterial agent in initial bacteria population	10
min_energy_growth_bacteria	The threshold of energy units to trigger division of a bacterial agent	10
aa_consumed	The number of amino acid units consumed by a bacterial agent per time step	1
aa_to_enegy_conversion	The number of gained units of energy per consumed unit of amino acid	2
vit_secreted	The number of vitamin units secreted by a bacterial agent per time step	1
secretion_cost_bacteria	The number of energy units lost per secreted vitamin unit	1
Mutant bacterial agents		
fit_loss	percentage of growth rate loss in mutants caused by a threefold increase in secretion (default: 0.1, corresponds to 10% lower growth rate/growth rate loss in mutants caused by a threefold increase in secretion (default: 0.1, corresponds to 10% lower growth rate than wild type)	0.1
increase_factor	The fold change in secretion of mutants in comparison to wild type bacteria (>1)	3
Yeast agents		
vit_consumed	The number of vitamin units consumed by a yeast agent per time step	8
aa_secreted	The number of amino acid units secreted by a yeast agent per time step	8
vit_to_enegy_conversion	The number of gained units of energy per consumed unit of vitamin	0.25
secretion_cost_yeast	The number of energy units lost per secreted amino acid unit	0.125
scale	value multiplied with min_energy_growth_bacteria to yield the respective division threshold for yeast	1
General settings		
birth_step	Determines how far away from each other (in units of patch size) daughter cells are positioned in the world after a division event	0.5
diffusion	Input for the NetLogo built in function *diffuse* to simulate diffusion. Determines the portion of the current amount of amino acid and vitamin in a patch that is in total distributed to all neighbouring patches.	0.5

mixing	values between 0 and 1. Determines how far (in units of patch size) each agent moves in each time step. This input is used as proxy for mixing strength.<br>(internal variable in Konstantinidis_ALE_loop.nlogo)	0.1
death_switch	Determines whether agents die when they run out of energy	ON
Initial conditions of patches		
initial_aa	The initial amount of amino acid units in each patch.	0
initial_vitamin	The initial amount of vitamin units in each patch.	0
Additional inputs in Konstantinidis_ALE_loop.nlogo		
number_transfers	number of transfers before simulation ends	25
number_runs	number of simulation repetitions for each set of inputs	200
