# Supplementary material for: Adaptive laboratory evolution of microbial co‐cultures for improved metabolite secretion
Source: Mol Syst Biol. 2021 Aug 9;17(8):e10189. doi: 10.15252/msb.202010189 (PMC8351387; doi:10.15252/msb.202010189)
Supplement: Supplementary file 2 — Expanded View Figures PDF [file MSB-17-e10189-s003.pdf]

Expanded View Figures

Figure EV1. Evaluation of traits associated with cell to cell interactions and the spatial structure of the co-cultures.

A Bacterial cell events in fluorescence-activated cell sorting (FACS) in yeast-lactic acid bacterial co-cultures. The co-cultures used are the same as in Fig 2C and collected at 84 h after inoculation ($n = 3$ biological replicates). The counts are normalized by the OD600 of the co-culture at the sample collection time. *P*-values based on unpaired two-tailed *t*-test.

B Yeast to bacterial cell ratio in co-cultures. Yeast cell numbers were estimated using RFP-positive events (showed in Fig 2D), and bacterial cell numbers were estimated using FACS as in A. ($n = 3$ biological replicates). *P*-values based on unpaired two-tailed *t*-test.

C Representative image of a fixed sample from the co-culture of the parental *L. plantarum* strain (Fig 2C) with *S. cerevisiae* (RFP positive), as seen in a wide-field microscope, during different time points of the growth kinetics. The scale bar in the bottom left corner of each microscope image is equal to 6 μm.

D Representative image of a fixed sample from the co-culture of the evolved *L. plantarum* isolate E6 (Fig 2C) with *S. cerevisiae* (RFP positive), as seen in a wide-field microscope, during different time points of the growth kinetics. The scale bar in the bottom left corner of each microscope image is equal to 6 μm.

E Bacterial cells in aqueous suspension (PBS) have the ability to attach to each other over time (auto-aggregation—left), and they exhibit a similar phenotype when they are mixed together with yeast cells (co-aggregation—right). $n = 3$ biological replicates; grey bars show mean ± SD.

F Estimation of the light absorbance at 570 nm from dye bound in the biofilm of bacterial monoculture (left), or bacterial co-culture with the parental auxotrophic yeast (right) Bar heights mark average; dots show individual data points; $n = 4$ biological replicates.

Source data are available online for this figure.

© 2021 The Authors

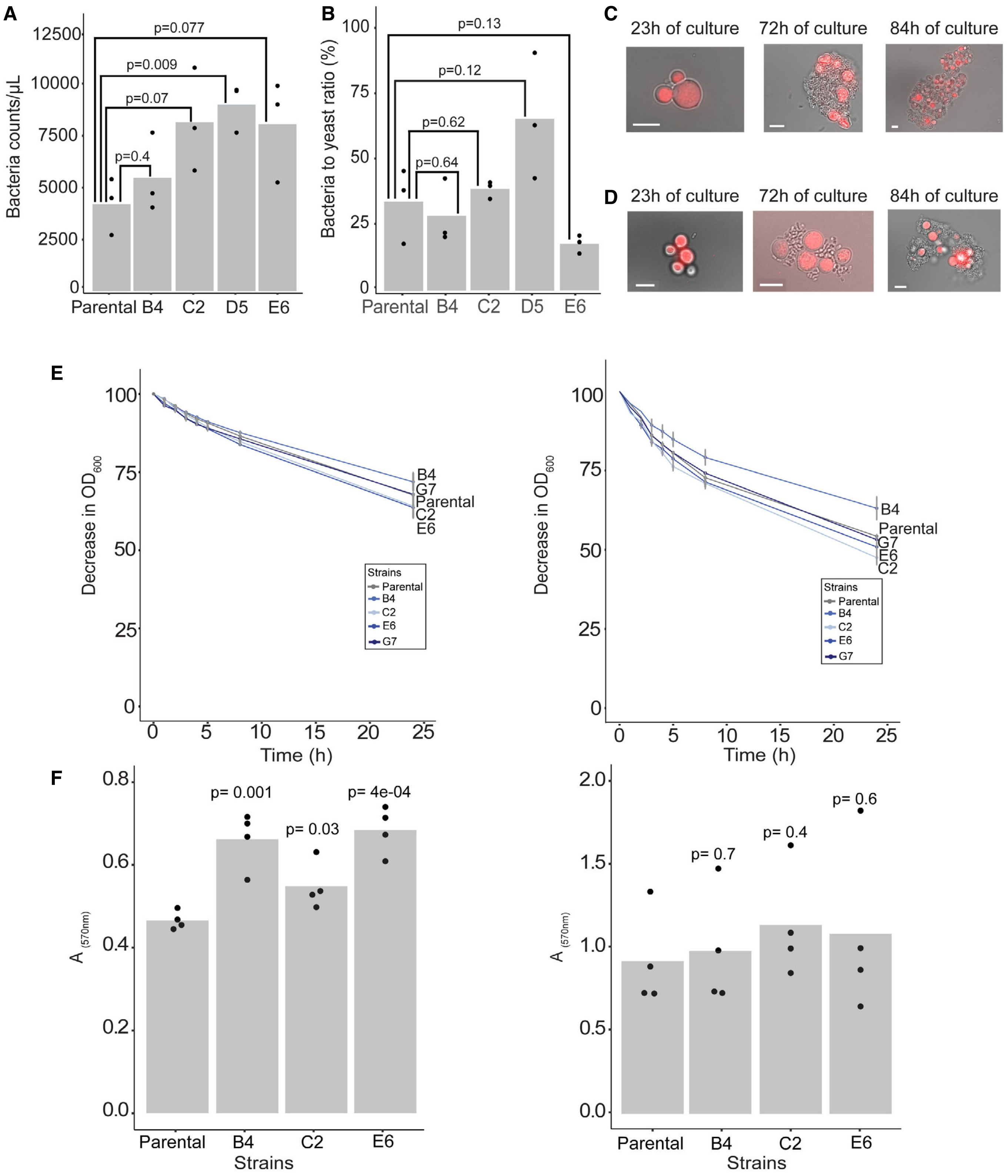

Figure EV1.

 © 2021 The Authors

Figure EV2. Effect of coevolution on growth fitness and amino acid secretion by *S. cerevisiae*.

A The growth rates and the maximum optical density reached by the parental *L. plantarum* strain cultured in the conditioned medium of the parental (Δrib4:rib5) yeast or the yeast isolates from the yeast-*L. plantarum* coevolution experiments. The vertical and the horizontal lines mark the average growth rate and optical density in the conditioned medium of the parental Δrib4:rib5 strain, respectively. *n* = 3 biological replicates.

B The growth rates and the maximum optical density reached by the evolved *L. plantarum* isolate E6 cultured in the conditioned medium of the parental (Δrib4:rib5) yeast or the yeast isolates from the yeast-*L. plantarum* coevolution experiments. The vertical and the horizontal lines mark the average growth rate and optical density in the conditioned medium of the parental Δrib4:rib5 strain, respectively. *n* = 3 biological replicates.

C–H Amino acid measurements (LC-MS analysis) of extracellular samples from the parental Δrib4:rib5 *S. cerevisiae* and 6 evolved yeast isolates. Shown are the areas under curve (AUC) from the corresponding peaks. Bar heights mark average; dots show individual data points; *n* = 3 biological replicates.

Source data are available online for this figure.

© 2021 The Authors

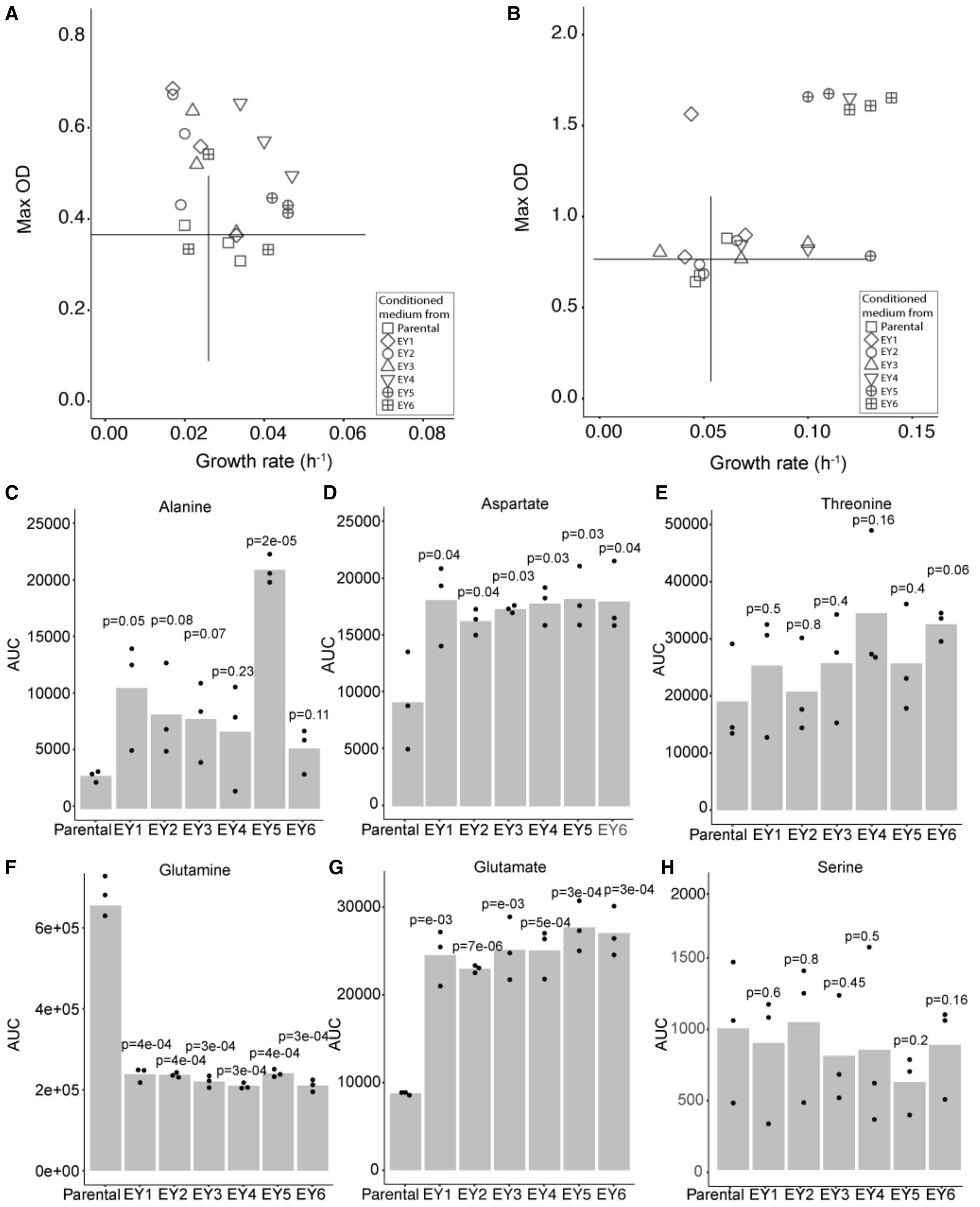

Figure EV2.

© 2021 The Authors

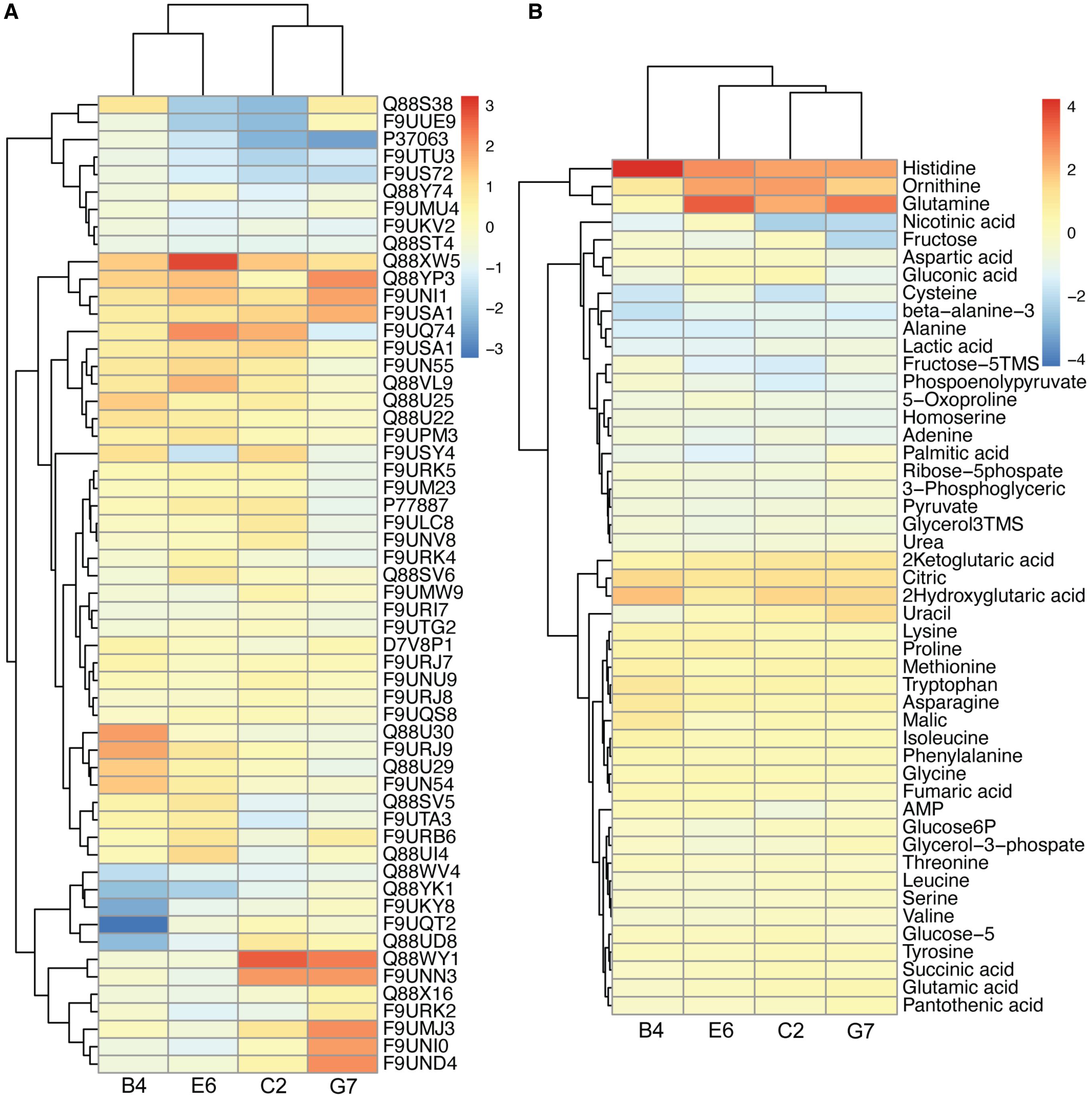

Figure EV3. Proteins and metabolites with altered abundance in evolved *L. plantarum*.

A, B Clustering analysis of proteins (A) and metabolites (B) with altered abundance in the evolved *L. plantarum* isolates. Hierarchical clustering was performed using Euclidean distance metric and complete linkage criterion. Colour gradient: log2 fold changes.

Source data are available online for this figure.

Figure EV4. Summary of the results of 200 simulation replicates of a 25-transfer *in silico* experiment.

A The simulation experiment was performed for four different secretion mutants (blue: 1x wild-type (parental) secretion level, red: 1.5× wild-type secretion level, yellow: 2× wild-type secretion level, purple: 3× wild-type secretion level) over several strengths of mixing (0–1). Percentage of successful mutant invasions (i.e. the mutant being present after 25 transfers). Each data point corresponds to frequency per 100 simulations.

B–E Summary statistics of the fraction of mutant bacteria in the total population of bacteria (mutant + wild type) for successful invasion events for each secretion mutant (colours as in panel A, mean: solid line, maximum: dashed line, minimum: dotted line). Error bars indicate one standard deviation (SD) around the mean. Error bars are only displayed if there were more than two values to calculate the SD from (more than two invasions in 200 simulations). A mean of zero indicates no successful invasion in 200 simulations.

Source data are available online for this figure.

 © 2021 The Authors

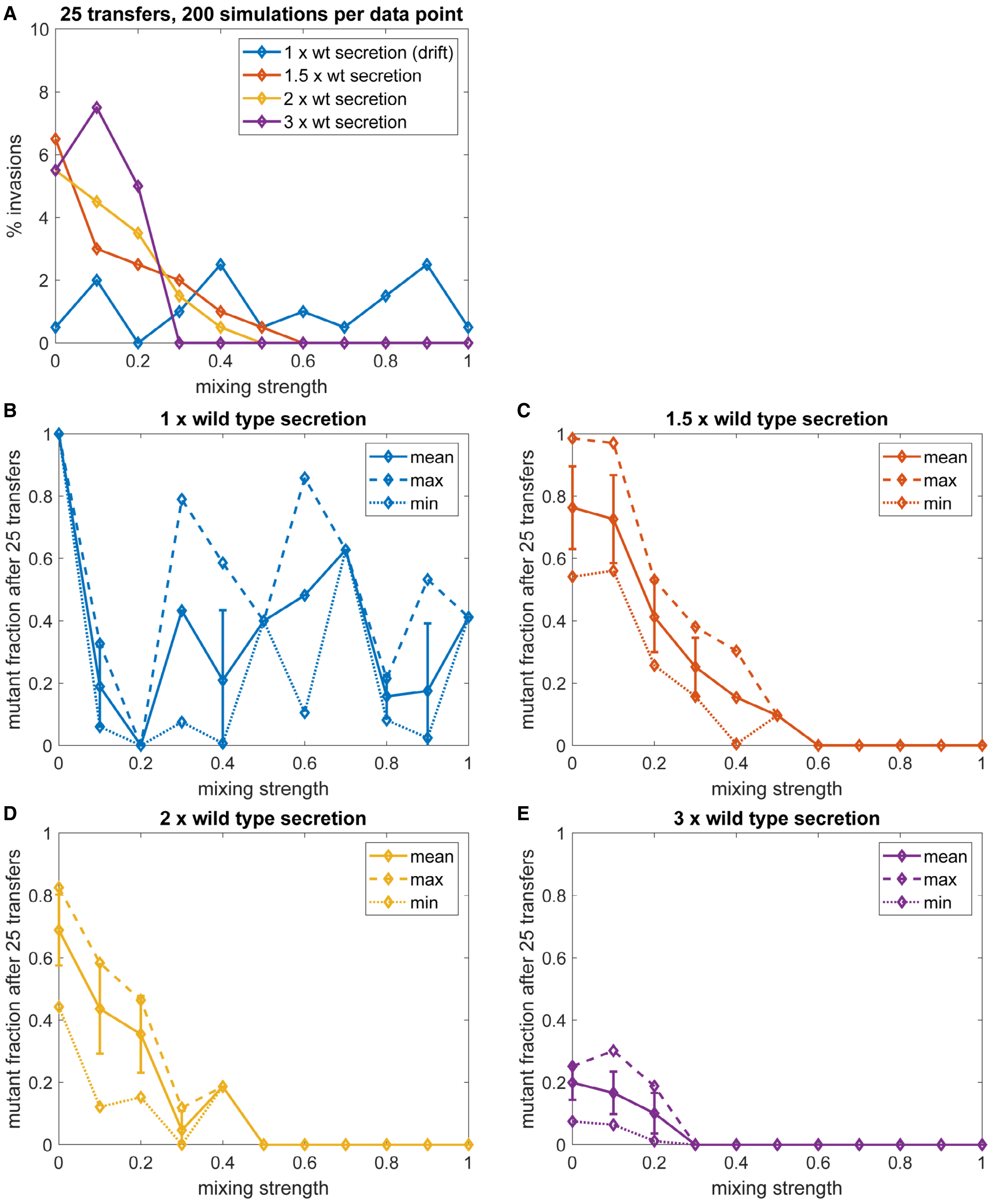
A
25 transfers, 200 simulations per data point
1 x wt secretion (drift)
1.5 x wt secretion
2 x wt secretion
3 x wt secretion
% invasions
mixing strength
B
1 x wild type secretion
C
1.5 x wild type secretion
D
2 x wild type secretion
E
3 x wild type secretion
mean
max
min
mutant fraction after 25 transfers
mixing strength

Figure EV4.

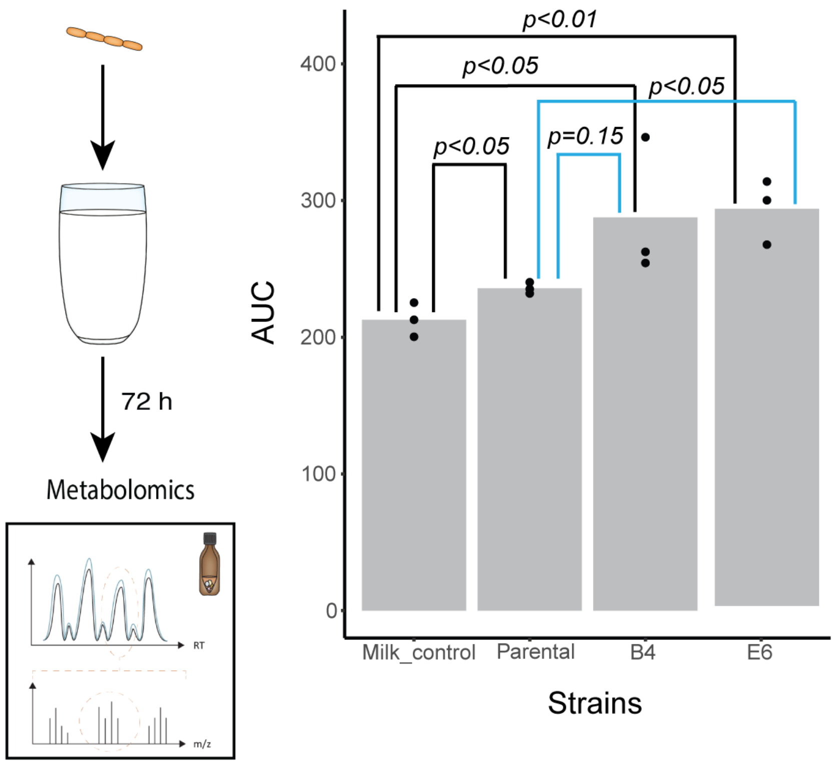

Figure EV5. Retention of improved riboflavin secretion phenotype in milk.

hRiboflavin levels (LC-MS analysis) in milk fermented by the parental *L. plantarum* and the evolved isolates B4 and E6. Bar heights mark average; dots show individual data points; $n = 3$ biological replicates. *P*-values based on unpaired two-tailed *t*-test.

Source data are available online for this figure.

 © 2021 The Authors
